# Supplementary material for: Effects of Diet and Lifestyle on Audio-Vestibular Dysfunction in the Elderly: A Literature Review
Source: Nutrients. 2022 Nov 8;14(22):4720. doi: 10.3390/nu14224720 (PMC9698578; doi:10.3390/nu14224720)
Supplement: Supplementary file 1 [file nutrients-14-04720-s001.zip › nutrients-1953378-supplementary.pdf]

Table S1. Newcastle-Ottawa Scale Quality Assessment of included Studies of the effects of diets on ARHL, ARVL and related components.

	Selection				Comparability		Outcome			
**First author/Year**	**Representativeness of the Exposed Cohort**	**Selection of the Non-Exposed Cohort**	**Ascertainment of Exposure**	**Outcome Was Not Present at Study Start**	**Comparability: Age and Sex**	**Comparability: Additional Factors**	**Assessment of Outcome**	**Was Follow-Up Long Enough for Outcomes to Occur? (>=3 month)**	**Adequacy of Follow -up of Cohorts (dropout <20%)**	**Total**
Gopinath/2010[72]		★	★	★	★		★		★	6
Curhan/2014[73]		★	★	★			★		★	5
Rosenhall/2015[58]	★		★	★	★		★		★	5
Kim/2015[75]	★		★	★	★	★	★	★	★	8
Dawes/2020[63]	★		★	★	★	★	★	★	★	8
Gopinath/2011[52]	★		★	★			★	★	★	6
Gopinath/2010[61]	★		★	★		★	★	★	★	7
Sardone/2020[50]	★		★	★		★	★	★	★	7
Lampignano/2021[93]			★	★		★	★	★	★	6
Tang/2021[101]	★		★	★			★	★	★	6
Durga/2007[124]	★	★	★	★	★		★	★	★	8
Gopinath/2011[135]	★		★	★		★	★		★	6
Kang/2014[126]	★		★	★	★	★	★		★	7
Lee/2018[145]	★		★	★			★		★	5

Table S2. Newcastle-Ottawa Scale Quality Assessment of included studies of the effects of lifestyles on ARHL, ARVL and related components.

	**Selection**				**Comparability**		**Outcome**			
**First author/Year**	**Representativeness of the Exposed Cohort**	**Selection of the Non-Exposed Cohort**	**Ascertainment of Exposure**	**Outcome Was Not Present at Study Start**	**Comparability: Age and Sex**	**Comparability: Additional Factors**	**Assessment of Outcome**	**Was Follow-Up Long Enough for Outcomes to Occur? (>=3 month)**	**Adequacy of Follow -up of Cohorts (dropout <20%)**	**Total**
Kawakami/2021[151]	★			★			★	★	★	5
Martines/2016[166]	★			★			★		★	4
Ekin/2016[168]		★	★	★			★		★	5
Jiang/2021[164]	★			★			★		★	4
Itoh/2001[174]		★	★			★			★	4
Ferrite & Santana/2005[173]			★			★	★		★	4
Pouryaghoub/2007[172]			★			★	★		★	4
Gopinath/2010[178]	★		★	★		★	★		★	6
Wada/2017[175]		★	★				★		★	4
Gopinath/2021[181]	★		★	★		★	★		★	6
